# Quantile Regression Analysis of the Distributional Effects of Air Pollution on Blood Pressure, Heart Rate Variability, Blood Lipids, and Biomarkers of Inflammation in Elderly American Men: The Normative Aging Study

**DOI:** 10.1289/ehp.1510044

**Published:** 2016-03-11

**Authors:** Marie-Abele Bind, Annette Peters, Petros Koutrakis, Brent Coull, Pantel Vokonas, Joel Schwartz

**Affiliations:** 1 Department of Statistics, Harvard University, Cambridge, Massachusetts, USA; 2 Institute of Epidemiology II, Helmholtz Zentrum München German Research Center for Environmental Health, Neuherberg, Germany; 3 Department of Environmental Health, and; 4 Department of Biostatistics, Harvard T.H. Chan School of Public Health, Boston, Massachusetts, USA; 5 VA Boston Healthcare System and the Department of Medicine, Boston University School of Medicine, Boston, Massachusetts, USA

## Abstract

**Background:**

Previous studies have observed associations between air pollution and heart disease. Susceptibility to air pollution effects has been examined mostly with a test of effect modification, but little evidence is available whether air pollution distorts cardiovascular risk factor distribution.

**Objectives:**

This paper aims to examine distributional and heterogeneous effects of air pollution on known cardiovascular biomarkers.

**Methods:**

A total of 1,112 men from the Normative Aging Study and residents of the greater Boston, Massachusetts, area with mean age of 69 years at baseline were included in this study during the period 1995–2013. We used quantile regression and random slope models to investigate distributional effects and heterogeneity in the traffic-related responses on blood pressure, heart rate variability, repolarization, lipids, and inflammation. We considered 28-day averaged exposure to particle number, PM2.5 black carbon, and PM2.5 mass concentrations (measured at a single monitor near the site of the study visits).

**Results:**

We observed some evidence suggesting distributional effects of traffic-related pollutants on systolic blood pressure, heart rate variability, corrected QT interval, low density lipoprotein (LDL) cholesterol, triglyceride, and intercellular adhesion molecule-1 (ICAM-1). For example, among participants with LDL cholesterol below 80 mg/dL, an interquartile range increase in PM2.5 black carbon exposure was associated with a 7-mg/dL (95% CI: 5, 10) increase in LDL cholesterol, while among subjects with LDL cholesterol levels close to 160 mg/dL, the same exposure was related to a 16-mg/dL (95% CI: 13, 20) increase in LDL cholesterol. We observed similar heterogeneous associations across low versus high percentiles of the LDL distribution for PM2.5 mass and particle number.

**Conclusions:**

These results suggest that air pollution distorts the distribution of cardiovascular risk factors, and that, for several outcomes, effects may be greatest among individuals who are already at high risk.

**Citation:**

Bind MA, Peters A, Koutrakis P, Coull B, Vokonas P, Schwartz J. 2016. Quantile regression analysis of the distributional effects of air pollution on blood pressure, heart rate variability, blood lipids, and biomarkers of inflammation in elderly American men: the Normative Aging Study. Environ Health Perspect 124:1189–1198; http://dx.doi.org/10.1289/ehp.1510044

## Introduction

Air pollution concentrations have been reduced in the past decades in the United States. However, ambient air pollution still causes adverse health outcomes at low concentrations below standards (Amancio and Nascimento 2014). Previous studies have shown evidence of heterogeneity in air pollution effects among individuals with different characteristics. Common analytic approaches to examine effect modification include the use of interaction terms (Bateson and Schwartz 2004; Breton et al. 2011; Hicken et al. 2013; Shumake et al. 2013; Yang et al. 2009) or the use of random slopes to examine between-subjects variability in air pollution estimates (Tager et al. 1998). However, these approaches have not provided sufficient understanding of how air pollution changes the shape of the distribution of risk factors or health outcomes. In particular, if larger effects were seen among people at the adverse end of such distributions, such findings would have important public health implications and would be quite important for health impact assessments. Investigating variations in air pollution effects based on the outcome of interest has received less attention but would address the issue of understanding changes in the distribution of risk.

Associations with air pollution can be estimated for individuals at different percentiles of the outcome distribution using quantile regression. The goal of this technique is to quantify the associations between exposure and specific quantiles of the outcome distribution, thereby allowing one to identify whether specific individuals with certain outcome levels are more affected by exposure. Hence, the use of quantile regression over the entire range of an outcome produces estimates that can be used to detect potential heterogeneity in exposure–outcome associations according to individual outcome levels. Another advantage of quantile regression is that it does not require assumptions about the distribution of the outcome (or the model residuals) and can therefore be used to estimate associations between air pollution and biomarkers of disease that are not normally distributed. An alternative approach, which is only available with repeated measures, is to fit random slopes for each subject and to use those slopes to examine heterogeneity of responses within the study population. In addition to requiring repeated measures per subject, this approach also makes assumptions about the distributions of the random slopes, typically assumed to be normal random variables with mean zero.

Using these approaches, we first aimed to examine whether air pollution distorts the distribution of established cardiovascular risk factors. Secondly, this study investigated whether air pollution associations with these cardiovascular risk factors vary by baseline individual levels of the same cardiovascular outcome, and whether those differences vary by pollutant. We investigated air pollution association on quantiles of blood pressure, heart rate variability, lipids, and inflammatory markers. We focused our investigation on elderly participants, who might be more susceptible to traffic-related air pollutants. We compared results from the quantile regression and random slopes approaches to evaluate the sensitivity of our conclusions to modeling assumptions.

## Methods

### Study Population

Participants included in this analysis were part of the Normative Aging Study (NAS), a longitudinal investigation established in Boston in 1963 by the U.S. Veterans Administration and limited to men (Bell et al. 1966). At the time of initial enrollment, participants were free of heart disease, hypertension, diabetes, cancer, recurrent asthma, or bronchitis. We measured cardiovascular-related outcomes on a total of 1,112 individuals one to seven times with intervals of 3–5 years (n_observations_ = 3,615) during the 1995–2013 period. The age range at baseline and over the entire study period was 49–97 years and 49–100 years, respectively. Medical visits included on-site physical examinations and detailed questionnaires after smoking abstinence and an overnight fast. Details of the methods and surveys are described elsewhere (Hu et al. 1996).

This study was approved by the Harvard School of Public Health and the Veteran Administration Institutional Review Boards (IRBs). Subjects provided written informed consent to participate in this study, which was approved by the Veteran Administration Central IRB.

### Air Pollution

Previous studies have suggested that the relevant exposure window for the association between between air pollution exposure and cardiovascular-related outcome ranges from hours to years (Brook et al. 2010; Devlin et al. 2014; Foraster et al. 2014; Rückerl et al. 2007). We chose to explore an intermediate-term exposure window, since it can serve as a median choice between short- and long-term windows. We *a priori* focused on air pollution concentrations measured during the 28-day period preceding each participant’s medical visit.

From 1995 onward, we measured ambient particle concentrations at the Harvard supersite located near downtown Boston and approximately 1 km from the medical center where the subjects were examined. We measured hourly particle number per cm^3^ (which captures fine and ultrafine particles with a 0.007–3 μm range in diameter) with a Condensation Particle Counter (TSI Inc, Model 3022A, Shoreview, MN, USA), hourly PM_2.5_ mass concentrations (particles ≤ 2.5 μm in diameter) using a Tapered Element Oscillation Microbalance (Model 1400A, Rupprecht and Pastashnick, East Greenbush, NY), and hourly PM_2.5_ black carbon (black carbon particles ≤ 2.5 μm in diameter) with an Aethalometer (Magee Scientific Co., Model AE-16, Berkeley, CA). A detailed description of the supersite has been previously published (Kang et al. 2010). Particle number measurements started in October 1999.

### Cardiovascular Outcomes

At each medical visit, we measured systolic blood pressure (SBP) and diastolic blood pressure (DBP) once in each arm while the subject was seated, using a standard cuff. We calculated the mean of right and left arm values and used it in these analyses.

In plasma, we measured plasma fibrinogen using a thrombin reagent called MDA Fibriquick, C-reactive protein concentrations using an immunoturbidimetric assay on the Hitachi 917 analyzer (Roche Diagnostics-Indianapolis, IN), and concentrations of intercellular adhesion molecule-1 (ICAM-1) and vascular cell adhesion molecule-1 (VCAM-1) using an enzyme-linked immunoabsorbent assay method (R&D Systems, Minneapolis, MN).

After a 5-min rest, we measured cardiac rhythm for 5–10 min in a sitting position with a two-channel electrocardiogram monitor using a sampling rate of 256 Hz per channel (Trillium 3000 model, Forest Medical, East Syracuse, NY). We obtained the standard deviation of normal-to-normal intervals (SDNN), low frequency (LF; 0.04–0.15 Hz), high frequency (HF; 0.15–0.4 Hz), and the logarithm of the LF:HF ratio with a fast Fourier transform using standard software (Trillium-3000, PC-Companion Software, Forest Medical). We measured QT interval from the QRS onset to the end of the T-wave only on normal or supraventricular beats. We calculated corrected QT values using the Bazett’s formula (Bednar et al. 2001), and the mean of corrected QT for the length of the recording as the outcome corresponding to each participant’s visit.

Before November 2000, we obtained serum concentrations of total cholesterol, high-density lipoprotein (HDL), and triglyceride using the BM/Hitachi 747-100 Automatic Analyzer (Roche Diagnostics Corporation, formerly Boehringer Mannheim Corp., IN). From November 2000 to December 2006, we used the Olympus AU640/AU400 Chemistry Analyzer (Olympus America Inc., PA), and from January 2006 to 2013, we used Abbott Architect assays (Abbott Diagnostics, IL). We calculated low-density lipoprotein (LDL) cholesterol in mg/dL using Friedewald’s formula (Friedewald et al. 1972):

LDL cholesterol = Total cholesterol – HDL cholesterol – (Triglyceride/5).

### Statistical Methods

We examined whether 28-day moving average air pollutant levels were associated with percentiles of the outcome distribution in the 10% increments (10th to 90th deciles). Because we measured each outcome of interest repeatedly for 77% of the participants, we fit quantile regressions for longitudinal data (Koenker 2004). Briefly, this method allows one to fit fixed-effects and correlated random-effects quantile regression models while relying on Bootstrap inference. We reported the quantile regression coefficients, scaled to correspond to differences in a given percentile of the outcome associated with an interquartile range (IQR) increase in the 28-day mean concentration of air pollution prior to the medical visit. We used the IQR because it reflects the spread of the distribution (i.e., 25th–75th percentiles) in the observed data.

Note that these differences are directly expressed in the outcome unit. We adjusted for the following potential confounders: temperature (24-hr mean of the day of the study visit and modeled continuously), relative humidity (24-hr mean of the day of the study visit and modeled continuously), as well as sine and cosine terms as a function of day of the season. We also controlled for time-varying factors likely to influence the outcome but not exposure such as: age (continuously modeled), physician-diagnosed diabetes (yes vs. no), body mass index (continuously modeled), smoking status (never vs. former vs. current), cumulative cigarette pack-years calculated for current and former smokers (continuously modeled), and statin use (current use vs. not). For blood pressure and heart rate variability, we adjusted for current use of antihypertensive medications (angiotensin-converting enzyme inhibitors, beta blockers, calcium channel blockers, angiotensin receptor blockers, and diuretics). For SDNN, we controlled for heart rate because standard deviation is likely to be larger as heart rate increases.

We assumed that the missing mechanism of the exposures happened completely at random and conducted complete case analyses. For instance, for particle number, our analysis is restricted to the period between October 1999 and February 2013, for which particle number measurements were obtained.

We assessed heterogeneity in the exposure–outcome association across quantiles of the outcome distribution using visual diagnostics of patterns of increasing or decreasing associations over the distribution. Because there can always be some variation due to noise in estimates from one decile to another, we relied on monotonic trends to detect potential real patterns of heterogeneity.

### Sensitivity Analyses

As secondary analyses, we fitted linear mixed-effects models with random intercepts and slopes for individual air pollutant effects to check for heterogeneous associations with the same outcomes of interest. Conditional on algorithm convergence, we obtained the subject-specific random slopes and calculated the individual effects (by adding the fixed and random effects) for participants with more than one visit to the VA clinic. Subsequently, we plotted these individual effects versus the outcome of interest measured at baseline.

## Results

Table 1 shows longitudinal characteristics of the population. Participants were all male, with a median age at baseline of 69 years old. At baseline, only 6% were current smokers, but a majority of the subjects were former smokers. Compared to subjects having a fewer number of medical visits, participants with more visits seemed healthier at the first medical visit (e.g., at the first medical visit, these participants were more likely to be never smokers, to not have diabetes, or to not take any medication). Characteristics of the outcomes at baseline and of the weather and air pollution during the study period are presented in Table 2 and Table 3, respectively. The estimates of IQR used as exposure increments in this analysis can be found in Table 3. While < 2% of observations were missing the concentrations of PM_2.5_ black carbon and PM_2.5_ mass, more than half of the observations were missing for particle number due to a delayed start of measurement.

**Table 1:** Demographic characteristics of the NAS participants by number of medical visits.

Visits	Age (in years) median	Cumulative smoking (pack-years^*a*^) median	Current statin user %	Obesity^*b*^ %	Medication^*c*^ %	Diabetic^*d*^ %	Smoking status		
							Never %	Former %	Current %
Baseline (*n* = 1,112)	69	14	18	25	47	10	28	66	6
Among participants having one visit (*n* = 259)
Visit 1	72	20	18	22	51	14	25	65	10
Among participants having two visit (*n* = 220)
Visit 1	72	13	22	25	53	10	28	66	6
Visit 2	76	13	37	29	64	15	28	67	5
Among participants having three visits (*n* = 147)
Visit 1	70	15	20	25	54	14	20	75	5
Visit 2	73	15	34	28	66	20	20	76	4
Visit 3	77	15	46	27	73	23	20	75	5
Among participants having four visits (*n* = 136)
Visit 1	70	11	17	28	46	10	32	60	8
Visit 2	73	11	32	29	60	13	32	62	6
Visit 3	77	11	47	29	68	16	31	65	4
Visit 4	80	11	57	24	80	20	30	68	2
Among participants having five visits (*n* = 178)
Visit 1	66	11	12	29	40	4	29	67	4
Visit 2	70	11	31	30	54	11	29	67	4
Visit 3	73	11	52	29	65	13	29	67	4
Visit 4	76	11	63	29	70	20	29	68	3
Visit 5	80	11	70	27	78	21	28	69	3
Among participants having six visits (*n* = 163)
Visit 1	64	10	16	23	37	3	32	64	4
Visit 2	67	10	31	29	42	6	32	65	3
Visit 3	70	10	42	23	53	8	32	66	2
Visit 4	73	10	55	24	62	12	32	66	2
Visit 5	76	10	63	23	69	15	32	66	2
Visit 6	80	10	62	21	75	18	32	67	1
Among participants having seven visits (*n* = 9)
Visit 1	65	0	0	22	33	0	56	44	0
Visit 2	68	0	11	33	44	0	56	44	0
Visit 3	70	0	22	22	56	0	56	44	0
Visit 4	74	0	22	22	56	0	56	44	0
Visit 5	76	0	44	22	56	11	56	44	0
Visit 6	78	0	56	22	56	11	56	44	0
Visit 7	81	0	67	22	56	11	56	44	0
^***a***^Pack-year is defined as the number of packs of cigarettes smoked per day times the number of years the person has smoked. ^***b***^Obesity status was defined as body mass index > 30 kg/m^2^. ^***c***^Current use of antihypertensive medications (angiotensin-converting enzyme inhibitors, beta blockers, calcium channel blockers, angiotensin receptor blockers, and diuretics). ^***d***^Diabetic status was diagnosed by a physician.									

**Table 2:** Baseline statistics of the cardiovascular-related outcomes of the 1,112 NAS participants.

Outcomes	Mean	5th Percentile	Median	95th Percentile
Blood pressure
Systolic (mmHg)	137	111	135	170
Diastolic (mmHg)	82	68	82	98
Heart rate variability and repolarization abnormality
Heart rate (beat/min)	65	47	65	85
SDNN (sec)	0.05	0.01	0.03	0.20
log_10_ (LF/HF)	–0.09	–1.06	–0.03	0.56
Corrected QT interval (msec)	386	313	384	459
Lipids (measured in serum)
HDL (mg/dL)	43.6	28	42	65
LDL (mg/dL)	145	86	144	205
Triglycerides (mg/dL)	152	61	129	308
Inflammation (measured in plasma)
Fibrinogen (mg/dL)	369	249	357	554
CRP (mg/L)	3.8	0.4	2.3	24.5
ICAM-1 (ng/mL)	291	156	270	533
VCAM-1 (ng/mL)	1,015	606	979	1,821
CRP, C-reactive protein.				

**Table 3:** Distributions of the weather and air pollution variables (1995–2013).

Variable	*n*_observations_^*a*^	*n*_missing_^*b*^	IQR	Percentiles		
				5th	50th	95th
Temperature (°C)
24-hr mean (day of the study visit)	3,606	9	14ºC	–3°C	13°C	25°C
Relative humidity (%)
24-hr mean (day of the study visit)	3,604	11	25%	41%	68%	92%
Particle number (number per cm^3^)
28-day mean (prior visit)	1,770	1,845^*c*^	13,845	8,651	17,874	41,629
Black carbon (μg/m^3^)
28-day mean (prior visit)	3,563	52	0.43	0.48	0.84	1.69
PM_2.5_ (μg/m^3^)
28-day mean (prior visit)	3,606	9	4.0	6.2	10.3	16.4
^***a***^Number of men = 1,112 and number of observations (study visits) = 3,615. ^***b***^Number of study visits with missing information. ^***c***^Measurements of particle number concentrations started in October 1999.						

**Figure 1:**
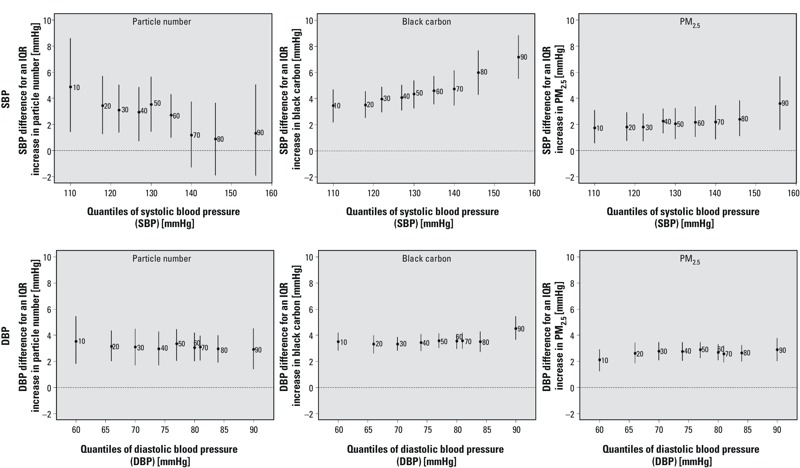
Associations between traffic-related air pollutants and quantiles of the distributions of SBP and DBP (adjusted for temperature, relative humidity, sine and cosine terms of the days of the season, age, physician-diagnosed diabetes, body mass index, smoking status, cumulative cigarette pack-years, current use of statin, and current use of antihypertensive medications). The *y*-axes represent the outcome difference (in the outcome unit) for an IQR increase in exposure. IQR for particle number = 13,845 number per cm^3^, IQR for PM_2.5_ black carbon = 0.43 μg/m^3^, and IQR for PM_2.5_ mass = 4.0 μg/m^3^. The numbers next to each point estimate indicate the deciles. Error bars represent 95% bootstrap CIs.

Our results showed that the associations between air pollution and blood pressure, heart rate variability, repolarization abnormality, lipids, and inflammation were generally not constant across quantiles. Figure 1 suggests increased blood pressure levels (fairly heterogeneous for SBP and fairly homogeneous for DBP) for all individuals in response to extended concentrations of particle number, black carbon, and PM_2.5_ mass. For instance, among participants with SBP > 155 mmHg (i.e., 90th percentile), an IQR increase in PM_2.5_ black carbon exposure was significantly associated with an increase of 7.2 mmHg [95% confidence interval (CI): 5.5, 8.8] in SBP, whereas among individuals with SBP around 110 mmHg (i.e., 10th percentile), an IQR increase in PM_2.5_ black carbon exposure was significantly associated with an increase of 3.5 mmHg (95% CI: 2.2, 4.7) in SBP. While the positive association between particle number and SBP was stronger in the lower quantiles of that outcome’s distribution (e.g., 10th percentile estimate = 4.9, 95% CI: 1.4, 8.6), the same association with PM_2.5_ black carbon and PM_2.5_ mass was stronger in the upper quantiles (e.g., 90th percentile estimate = 3.6, 95% CI: 1.6, 5.7).

**Figure 2:**
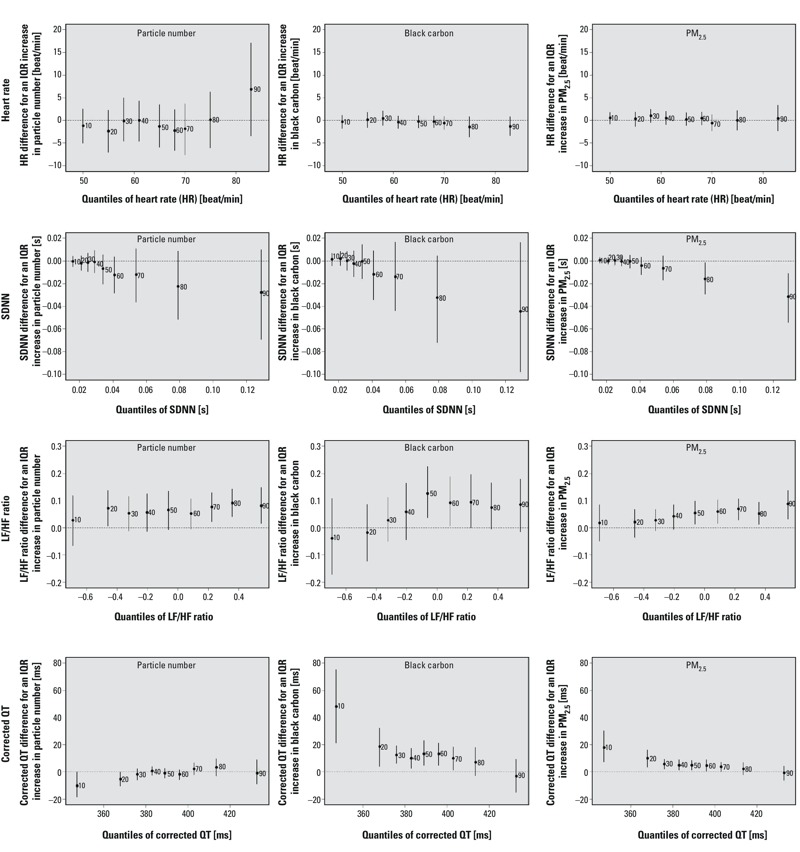
Associations between traffic-related air pollutants and quantiles of the distributions of heart rate, SDNN, log(LF:HF ratio), and corrected QT interval (adjusted for temperature, relative humidity, sine and cosine terms of the days of the season, age, physician-diagnosed diabetes, body mass index, smoking status, cumulative cigarette pack-years, current use of statin, and current use of antihypertensive medications). For SDNN, we also controlled for heart rate because standard deviation is likely to be larger as heart rate increases. The *y*-axes represent the outcome difference (in the outcome unit) for an IQR increase in exposure. IQR for particle number = 13,845 number per cm^3^, IQR for PM_2.5_ black carbon = 0.43 μg/m^3^, and IQR for PM_2.5_ mass = 4.0 μg/m^3^. The numbers next to each point estimate indicate the deciles. Error bars represent 95% bootstrap CIs. Note: ms, millisecond; s, second.

Figure 2 indicates that among individuals with SDNN > 0.08 sec (i.e., 80th percentile), an IQR increase in PM_2.5_ mass exposure was significantly related to a decrease of 0.016 sec (95% CI: –0.030, –0.001) or more in SDNN, and that among particpants with SDNN around 0.02 sec (i.e., 20th percentile), PM_2.5_ mass exposure was not significantly associated with SDNN (20th percentile estimate = –0.0002, 95% CI: –0.003, 0.003). We observed significant positive associations of PM_2.5_ black carbon and PM_2.5_ mass with corrected QT interval mostly in individuals with corrected QT interval < 380 msec (i.e., between the 30th and 40th percentiles). Among individuals with corrected QT lower than 360 msec (i.e., 10th percentile), an IQR increase in PM_2.5_ black carbon exposure was significantly related to an increase of 48 msec (95% CI: 21, 75) in corrected QT, but PM_2.5_ black carbon exposure was not significantly associated with corrected QT among participants with corrected QT exceeding 420 msec (90th percentile estimate = –3, 95% CI: –15, 9). We did not observe any obvious heterogeneity in the exposure–outcome association across the distributions of heart rate and the LH:HF ratio, except for the positive association between PM_2.5_ black carbon and LF:HF ratio (that was observed among individuals above the median (median ≈ –0.1) of the LF:HF ratio distribution).

**Figure 3:**
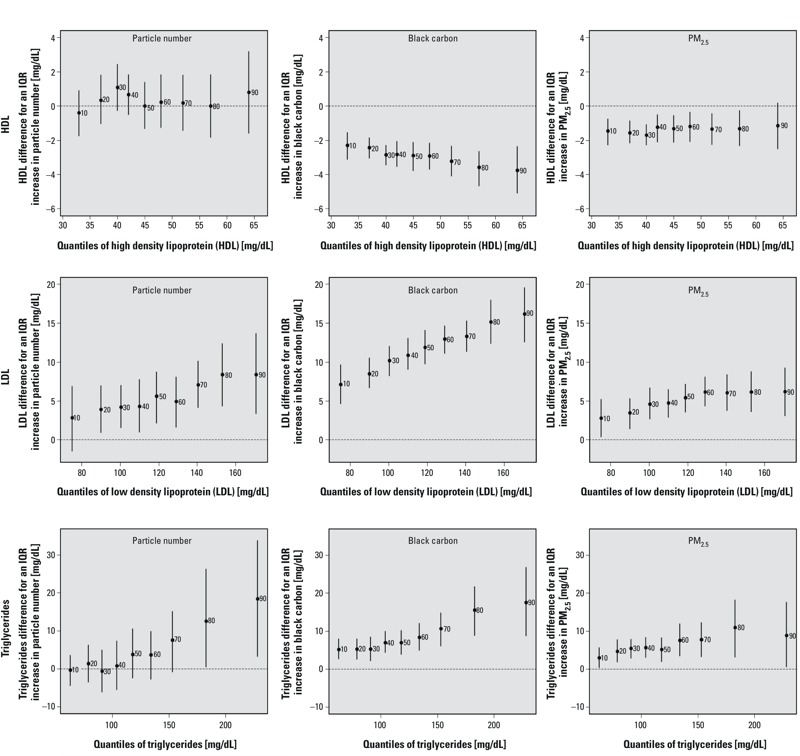
Associations between traffic-related air pollutants and quantiles of the distributions of HDL cholesterol, LDL cholesterol, and triglycerides (adjusted for temperature, relative humidity, sine and cosine terms of the days of the season, age, physician-diagnosed diabetes, body mass index, smoking status, cumulative cigarette pack-years, and current use of statin). The *y*-axes represent the outcome difference (in the outcome unit) for an IQR increase in exposure. IQR for particle number = 13,845 number per cm^3^, IQR for PM_2.5_ black carbon = 0.43 μg/m^3^, and IQR for PM_2.5_ mass = 4.0 μg/m^3^. The numbers next to each point estimate indicate the deciles. Error bars represent 95% bootstrap CIs.

Figure 3 also suggests some heterogeneity in the air pollution–lipid association across deciles of the lipid distributions. For example, among participants with LDL cholesterol < 80 mg/dL (i.e., 10th percentile), an IQR increase in PM_2.5_ black carbon exposure was associated with a 7 mg/dL (95% CI: 5, 10) increase in LDL cholesterol, whereas among subjects with LDL cholesterol levels close to 160 mg/dL (i.e., 90th percentile), the same exposure was related to a 16 mg/dL (95% CI: 13, 20) increase in LDL cholesterol. Moreover, while the negative PM_2.5_ black carbon–HDL cholesterol association was stronger for individuals with HDL levels > 50 mg/dL (i.e., between the 60th and 70th percentiles), the associations between all air pollutants of interest in this study were stronger at the highest percentiles of the triglycerides distribution.

**Figure 4:**
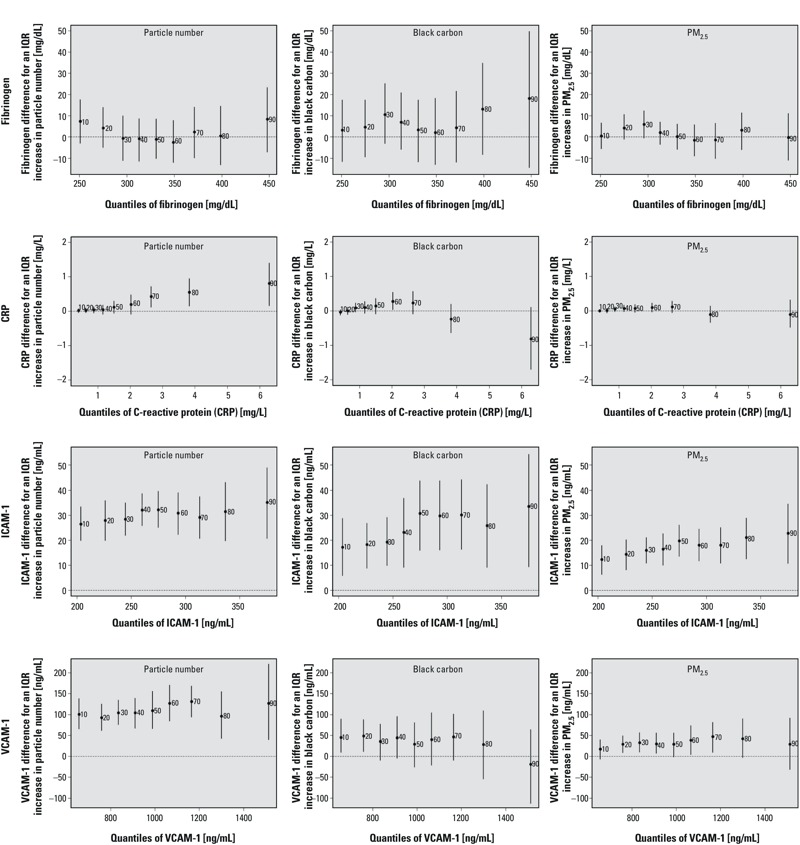
Associations between traffic-related air pollutants and quantiles of the distributions of fibrinogen, C-reactive protein, ICAM-1, and VCAM-1 (adjusted for temperature, relative humidity, sine and cosine terms of the days of the season, age, physician-diagnosed diabetes, body mass index, smoking status, cumulative cigarette pack-years, and current use of statin). The *y*-axes represent the outcome difference (in the outcome unit) for an IQR increase in exposure. IQR for particle number = 13,845 number per cm^3^, IQR for PM_2.5_ black carbon = 0.43 μg/m^3^, and IQR for PM_2.5_ mass = 4.0 μg/m^3^. The numbers next to each point estimate indicate the deciles. Error bars represent 95% bootstrap CIs.

Figure 4 exhibits fairly homogenous air pollution–inflammation associations (i.e., no meaningful monotone patterns across quantiles of fibrinogen and VCAM-1). However, for participants with C-reactive levels > 2 mg/L (i.e., 60th percentile), an IQR increase in particle number was associated with a 0.4 mg/L (95% CI: 0.1, 0.7) increase or more in C-reactive protein, while we observed null associations for the 10th to 60th quantiles. In addition, while an IQR increase in PM_2.5_ mass was associated with a 12-ng/mL (95% CI: 6, 18) increase in the 10th percentile of the ICAM-1 distribution (corresponding to 200 ng/mL), it was associated with a 23-ng/mL (95% CI: 11, 34) increase in the 90th percentile (corresponding to 375 ng/mL).

We noted that the quantile regression coefficients tend to have greater estimated variance when estimated at the tails of the distributions, which may be due to a fewer number of observations at the tails (compared to the center) used in the quantile regression.

### Sensitivity Analyses

As secondary analyses, we assessed the associations between baseline level of risk factors and individual effects estimates (obtained by mixed-effects models). These analyses included the subset of men with more than one medical visit (i.e., 77% of the study population).

Similarly as our results in Figure 1 (i.e., increasing black carbon–SBP associations and decreasing particle number–SBP associations), the positive effects of PM_2.5_ black carbon (and PM_2.5_ mass) on SBP appeared to be stronger among participants with higher SBP measured at baseline, while the particle number–SBP association appeared to be stronger for participants with lower SBP at baseline (see Figure S1).

The mixed-effects model did not converge when estimating the association between heart rate and particle number (due to missing data for particle number), but results suggest stronger negative effects of PM_2.5_ black carbon and PM_2.5_ mass among participants with lower baseline heart rates (see Figure S2), in contrast with quantile regression estimates that were relatively flat over the heart rate distribution (Figure 2). Consistent with the quantile regression results, stronger negative associations were estimated for all of the air pollutants among participants with higher baseline SDNN. Both analyses also suggested stronger positive associations of PM_2.5_ black carbon and PM_2.5_ mass with corrected QT intervals among those with lower baseline corrected QT interval. However, while the quantile regression suggested no association with particle number other than a negative association among those with the lowest corrected QT interval (Figure 2), the estimates from the mixed-effects model approach suggested stronger positive associations as baseline QT interval increased (see Figure S2). While the air pollution–LH:HF ratio association was fairly homogenous across quantiles (Figure 2), the mixed-effects model suggested stronger positive associations between all pollutants and the log LH:HF ratio among those with higher baseline log LH:HF ratio.

Stronger positive associations with LDL cholesterol and triglycerides were estimated for all examined air pollutants among individuals with higher baseline levels (see Figure S3), consistent with the quantile regression results (Figure 3). However, the mixed-effects models suggested positive associations of particle number with HDL that were stronger as baseline HDL increased, whereas quantile regression did not suggest a consistent pattern of associations between particle number and HDL over the HDL distribution. In addition, while quantile regression suggested that the negative association between PM_2.5_ black carbon and HDL was stronger among those with higher HDL levels (Figure 3), estimates from the mixed-effects model did not suggest a consistent pattern of associations according to baseline HDL.

Although air pollution–fibrinogen associations from quantile regressions did not show consistent increases or decreases along the fibrinogen distribution (Figure 4), the mixed-effects model estimates suggested stronger associations with particle number and PM_2.5_ black carbon among participants with higher baseline fibrinogen (see Figure S4). In contrast, mixed-effects model estimates suggested that associations between fibrinogen and PM_2.5_ mass were strongest among those with the lowest baseline fibrinogen levels. The mixed-effects model did not suggest variation in associations between particle number and C-reactive protein (see Figure S4), in contrast with a pattern of stronger associations among those with higher C-reactive protein concentrations based on quantile regression (Figure 4). The mixed-effects models also suggested stronger associations between VCAM-1 and all three air pollutants (especially PM_2.5_ black carbon and PM_2.5_ mass) in contrast with relatively consistent associations across the distribution based on quantile regression (though PM_2.5_ black carbon did show positive associations at the low end of the distribution only). Associations with ICAM-1 were stronger for higher baseline exposures based on both approaches, though the patterns appear much more pronounced for mixed-effects estimates.

## Discussion

Our findings add further support for effects of ambient particulate air pollution on known cardiovascular risk factors (i.e., SBP, heart rate variability, repolarization abnormality, lipids, and inflammation). For those outcomes, we found evidence that the air pollution association is not merely a shift in the distribution of the biomarkers in an adverse direction, but a change in the distribution across the population. These associations are missed when standard regression techniques are applied. In particular, associations were often stronger among individuals whose biomarker levels already suggested higher risks. For example, the association between PM_2.5_ black carbon and LDL cholesterol was strongest in men with LDL concentrations > 140 mg/dL, and the association between PM_2.5_ black carbon and SBP was strongest in men with SBP > 140 mmHg.

Findings were not always consistent between the two approaches (i.e., differential quantile regression coefficients along the outcome distribution and differential individual associations by baseline outcome level using mixed-effects models with subject-specific random intercepts and slopes). For example, the subject-specific associations for PM_2.5_ black carbon and PM_2.5_ mass were higher in participants with higher SBP measured at baseline. However, this approach cannot be used when there are no repeated measures, whereas quantile regression can. That is, because random slope models assume a normal distribution of the subject-specific slopes about the population mean. In contrast, some of the findings from the quantile models suggest that the distribution is quite skewed and thus the normality assumption does not hold (e.g., the association between particle number and C-reactive protein does not appear to be centered around the population mean, but the association has a long upper tail, Figure 4). This is because the response is null except for participants at one extreme of the distribution of baseline outcomes. The violation of the normality assumption could explain some of the inconsistencies between the two approaches.

### Previous Evidence on Shifts in Changes of Risk Factor Distributions

A previous study has reported a shift in the heart rate distribution due to an air pollution episode in 1985 in Central Europe (Peters et al. 2000). The authors found no obvious distributional distortions on heart rate when comparing air pollution episode to non-episode, which is consistent with our analysis that found no evidence against homogeneous associations along the heart rate distribution. Our quantile regression results are also directionally fairly consistent with mean regression analyses investigating the same cardiovascular outcomes either in the same cohort (Mordukhovich et al. 2009; Ren et al. 2010; Zeka et al. 2006), or in previous studies (Hampel et al. 2010; Hoffmann et al. 2012; Peters et al. 1999; Rückerl et al. 2007), but capture additional shifts in the distribution. For instance, in the same NAS cohort, exposure to PM_2.5_ black carbon (7-day moving average exposure to PM_2.5_ black carbon) was associated with increased SBP and DBP (Mordukhovich et al. 2009). While this previous study did not find any association between PM_2.5_ mass and mean blood pressure, our quantile regression analysis (including more recent data) revealed associations between PM_2.5_ mass and increased SBP and DBP along the entire distributions. An important feature of quantile regression is that the effect estimate is expressed in mmHg and thus can be directly clinically interpretable unlike studies analyzing log-transformed outcome data. Moreover, in an experimental study that examined healthy and asthmatic volunteers, Gong et al. (2004) also reported a decrease in SDNN associated with controlled exposures to ambient coarse particles. Previous studies have identified heterogeneity in the association between air pollution and cardiovascular outcomes based on risk factors such obesity and diabetic status (Baja et al. 2010), high viscosity (Peters et al. 2000), psychological factors (Madrigano et al. 2012), temperature (Ren et al. 2011), genetic variants (Ljungman et al. 2009; Park et al. 2006; Ren et al. 2010; Wilker et al. 2010), and epigenetic changes (Bind et al. 2012). In this study, we observed disparities based on outcome levels, which is a useful summary of multiple vulnerability cardiovascular risk factors (for this population of elderly white men).

### Variation among Air Pollution Exposures

While PM_2.5_ black carbon was positively correlated with PM_2.5_ mass (Spearman correlation = 0.77), particle number was not correlated with PM_2.5_ black carbon or PM_2.5_ mass (Spearman correlation = –0.07 and Spearman correlation = 0.07, respectively).

Quantile regression allowed us to identify evidence of effects on the overall shape of the outcome distribution, rather than shifts in the population mean only. For example, particle number concentration was positively associated with SBP among men with SBP in the lower percentiles of the distribution, but not among men with higher SBP. This suggests that exposure to higher particle number concentrations will shift the left tail of the distribution of SBP toward the mean, without altering SBP of participants in the upper tail of the distribution. In contrast, the association between PM_2.5_ black carbon and SBP was positive for all men, but strongest among men with higher SBP, suggesting a larger shift in the upper tail than the lower tail of the SBP distribution. While both are particles from traffic, particle number concentration represents the concentration of ultrafine and fine particles between 0.007 and 0.300 μm, including ultrafine particles that are freshly generated, whereas PM_2.5_ black carbon particles are a mix of freshly generated ultrafine particles (aerodynamic particle diameter size d_a_ < 0.1 μm) and aged traffic particles (mostly in the accumulation mode, 0.1 < d_a_ < 1.0 μm) (Kang et al. 2010). Different types of particles may therefore affect certain parts of the SBP distribution differently. This finding provides evidence that different biological mechanisms may be involved in the adverse responses induced by fine and ultrafine particles. Finally, we did not observe any monotonically increasing or decreasing pattern in the associations between traffic-related particles and DBP across the distribution of this outcome. Generally, associations between the three particle metrics investigated in this study and DBP were fairly homogenous across the DBP distribution.

This quantile analysis reveals some association between PM_2.5_ mass and the upper tail of the SDNN distribution but no association between PM_2.5_ mass and the lower tail of the distribution. In addition, we observed positive associations between PM_2.5_ mass and the higher percentiles of the log(LF:HF) ratio distribution, indicating that the mean effect was driven by the highest percentiles of the distribution. For PM_2.5_ mass, SDNN was reduced and the log(LF:HF) ratio was increased at the higher end of their distribution. This result suggests a health effect of PM_2.5_ mass involving a decrease in high frequencies, and thus points toward a potential impact on the parasympathetic pathway. For corrected QT interval, our results suggest that participants with low corrected QT interval were susceptible to increases in this outcome due to exposure of PM_2.5_ black carbon and PM_2.5_ mass. We also observed negative associations between particle number and the lowest quantiles of the corrected QT interval, which was opposite to that found for PM_2.5_ mass.

Our findings for lipids suggest that for high traffic-related exposures the right-tail of the LDL cholesterol and triglycerides distributions became longer with increases in exposure, again indicating that participants already at higher risk were impacted more. For HDL cholesterol, results from both statistical approaches (i.e., quantile regression and mixed-effects model) were not consistent. The main inconsistency was found with particle number and HDL cholesterol. While the quantile regression approach suggested homogenous particle number–HDL cholesterol associations along the HDL cholesterol distribution, the mixed-effects model approach indicated heterogeneity in the individual responses according to baseline HDL cholesterol level.

Particle number was associated with C-reactive protein only at the highest percentiles of the distribution; suggesting an effect in participants who already had elevated C-reactive protein levels. That is, results suggest that the right tail of the C-reactive protein distribution is extended by exposure to particle number concentration, similarly as what was observed for IFN-γ DNA methylation in the same cohort (Bind et al. 2015). High levels of C-reactive protein have been related to cardiovascular disease (Ridker et al. 2010). Hence, this may suggest that individuals with higher risk of inflammation and cardiovascular disease may be the ones primarily being affected by exposure to particle number concentration. However, this result was not confirmed by the secondary analysis, which highlighted few participants at the extreme tail of the C-reactive protein distribution. For participants who already had high levels of ICAM-1, the effect of PM_2.5_ mass exposures on ICAM-1 was almost doubled (compared to individuals with lower ICAM-1 levels). This result demonstrates that in the presence of effect heterogeneity across the distribution of an outcome it is not adequate to report the mean estimate because it summarizes these effect estimate that differ across the range of the distribution, including those with opposing signs.

### Strengths and Limitations

Unlike mean regression analysis, the statistical approach using quantile regression is distribution free; thus, no transformation of the outcome is necessary. Estimates from quantile regression can therefore be directly expressed in the unit of the outcome of interest and provide clinically interpretable health impact. This method may capture associations that occur only at the tails of the distribution and might be otherwise missed. Another advantage of the quantile regression is that it captures distributional distortion. Finally, using both methods (quantile regression and mixed-effects model), we could sometimes demonstrate fairly similar heterogeneous effects of traffic-related air pollutants, but sometimes obtain inconsistent results (possibly due to the difference in methods and their associated assumptions).

One limitation of our study is the use of a single air pollution monitoring site. Since the study participants lived in the greater Boston, Massachusetts, area with a median distance of about 20 km, we assumed that the ambient air pollutant concentrations measured at the central monitoring site could serve as surrogates of their exposures. We also assumed the measurement error of the air pollutants concentrations to be primarily a Berkson measurement error. A previous study supports the assumption of this measurement error for air pollution exposures assessed at a central site (Zeger et al. 2000). Two studies support the use of exposure measured at a central monitoring site (e.g., for PM_2.5_ and PM_10_) in epidemiological studies (Alexeeff et al. 2015; Janssen et al. 1998). Whether these findings are generalizable to the present study would partly depend on where the studies were conducted. However, correction for measurement error may yield less biased estimates for spatially heterogeneous air pollutants, such as black carbon and particle number. The proportion of missing measurements for particle number is also relatively high, mostly due to the fact that measurement started later than for the other air pollutants. The results for this exposure, therefore, relates to a different study period. This study period likely had lower levels over the full study period, as pollution levels have been declining steadily in the New England region. Therefore, ultimately this loss of data could be expected to reduce our power to detect associations with this exposure, but note we still detect associations with particle number.

We focused on intermediate-term associations with traffic-related air pollution (i.e., using 28-day moving average), and it could be the case that other exposure time windows are more relevant to these outcomes. In addition, this longitudinal design following an elderly cohort is subject to loss of follow-up. Therefore, the studied population constitutes a healthier subset of the population for later visits.

## Conclusions

Our results suggest that air pollution distorts the distribution of established cardiovascular risk factors, and provides evidence that, in many cases, effects may be more pronounced in men who are already at increased risk of cardiovascular disease. More thorough preventive measures are required for individuals who are chronically exposed to high levels of particulate matter air pollution. Future studies could investigate whether these findings generalize to younger or female populations and to different ethnicities. Moreover, quantile regression may be used to obtain more accurate risk assessments and should be considered in environmental epidemiology investigations.

## Supplemental Material

(2.5 MB) PDF
