# Supplementary material for: Quantile Regression Analysis of the Distributional Effects of Air Pollution on Blood Pressure, Heart Rate Variability, Blood Lipids, and Biomarkers of Inflammation in Elderly American Men: The Normative Aging Study
Source: Environ Health Perspect. 2016 Mar 11;124(8):1189–98. doi: 10.1289/ehp.1510044 (PMC4977045; doi:10.1289/ehp.1510044)
Supplement: (2.5 MB) PDF [file ehp.1510044.s001.acco.pdf]

Environ Health Perspect
DOI: 10.1289/ehp.1510044

Note to readers with disabilities: *EHP* strives to ensure that all journal content is accessible to all readers. However, some figures and Supplemental Material published in *EHP* articles may not conform to 508 standards due to the complexity of the information being presented. If you need assistance accessing journal content, please contact ehp508@niehs.nih.gov. Our staff will work with you to assess and meet your accessibility needs within 3 working days.

Supplemental Material

Quantile Regression Analysis of the Distributional Effects of Air Pollution on Blood Pressure, Heart Rate Variability, Blood Lipids, and Biomarkers of Inflammation in Elderly American Men: The Normative Aging Study

Marie-Abele Bind, Annette Peters, Petros Koutrakis, Brent Coull, Pantel Vokonas, and Joel Schwartz

Table of Contents

Figure S1. Individual effects of traffic-related air pollutants on systolic blood pressure and diastolic blood pressure versus baseline systolic blood pressure and baseline diastolic blood pressure, respectively. We fit linear mixed-effects models with random intercepts and slopes for individual air pollutant effects to check for heterogeneous associations with the same outcomes of interest. We adjusted for temperature, relative humidity, sine and cosine terms of the days of the season, age, physician-diagnosed diabetes, body mass index, smoking status, cumulative cigarette pack-years, current use of statin, and current use of antihypertensive medications. Conditional on algorithm convergence, we obtained the subject-specific random slopes and calculated the individual effects (by adding the fixed and random effects) for participants with more than one visit. Subsequently, we plotted these individual effects (that correspond to individual increases in the outcome for an interquartile range increase in exposure) versus the

outcome of interest measured at baseline. The units of the y-axes correspond the units of the outcome. Estimates are limited to participants with more than one study visit.

Figure S2. Individual effects of traffic-related air pollutants on heart rate, SDNN, log(LF/HF), and corrected QT interval versus baseline heart rate, baseline SDNN, baseline log(LF/HF), and baseline corrected QT interval, respectively. We fit linear mixed-effects models with random intercepts and slopes for individual air pollutant effects to check for heterogeneous associations with the same outcomes of interest. We adjusted for temperature, relative humidity, sine and cosine terms of the days of the season, age, physician-diagnosed diabetes, body mass index, smoking status, cumulative cigarette pack-years, current use of statin, and current use of antihypertensive medications. For SDNN, we also controlled for heart rate because standard deviation is likely to be larger as heart rate increases. Conditional on algorithm convergence, we obtained the subject-specific random slopes and calculated the individual effects (by adding the fixed and random effects) for participants with more than one visit. Subsequently, we plotted these individual effects (that correspond to individual increases in the outcome for an interquartile range increase in exposure) versus the outcome of interest measured at baseline. The units of the y-axes correspond the units of the outcome. Estimates are limited to participants with more than one study visit.

Figure S3. Individual effects of traffic-related air pollutants on HDL cholesterol, LDL cholesterol, and triglyceride versus baseline HDL cholesterol, baseline LDL cholesterol and baseline triglyceride, respectively. We fit linear mixed-effects models with random intercepts and slopes for individual air pollutant effects to check for heterogeneous associations with the same outcomes of interest. We adjusted for temperature, relative humidity, sine and cosine terms of the days of the season, age, physician-diagnosed diabetes, body mass index, smoking status, cumulative cigarette pack-years, and current use of statin. Conditional on algorithm convergence, we obtained the subject-specific random slopes and calculated the individual effects (by adding the fixed and random effects) for participants with more than one visit. Subsequently, we plotted these individual effects (that correspond to individual increases in the outcome for an interquartile range increase in exposure) versus the outcome of interest measured at baseline. The units of the y-axes correspond the units of the outcome. Estimates are limited to participants with more than one study visit.

Figure S4. Individual effects of traffic-related air pollutants on fibrinogen, C-reactive protein, ICAM-1, and VCAM-1 versus baseline fibrinogen, baseline C-reactive protein, baseline ICAM-1, and baseline VCAM-1, respectively. We fit linear mixed-effects models with random intercepts and slopes for individual air pollutant effects to check for heterogeneous associations with the same outcomes of interest. We adjusted for temperature, relative humidity, sine and cosine terms of the days of the season, age, physician-diagnosed diabetes, body mass index, smoking status, cumulative cigarette pack-years, and current use of statin. Conditional on algorithm convergence, we obtained the subject-specific random slopes and calculated the individual effects (by adding the fixed and random effects) for participants with more than one visit. Subsequently, we plotted these individual effects (that correspond to individual increases in the outcome for an interquartile range increase in exposure) versus the outcome of interest measured at baseline. The units of the y-axes correspond the units of the outcome. Estimates are limited to participants with more than one study visit.

Figure S1

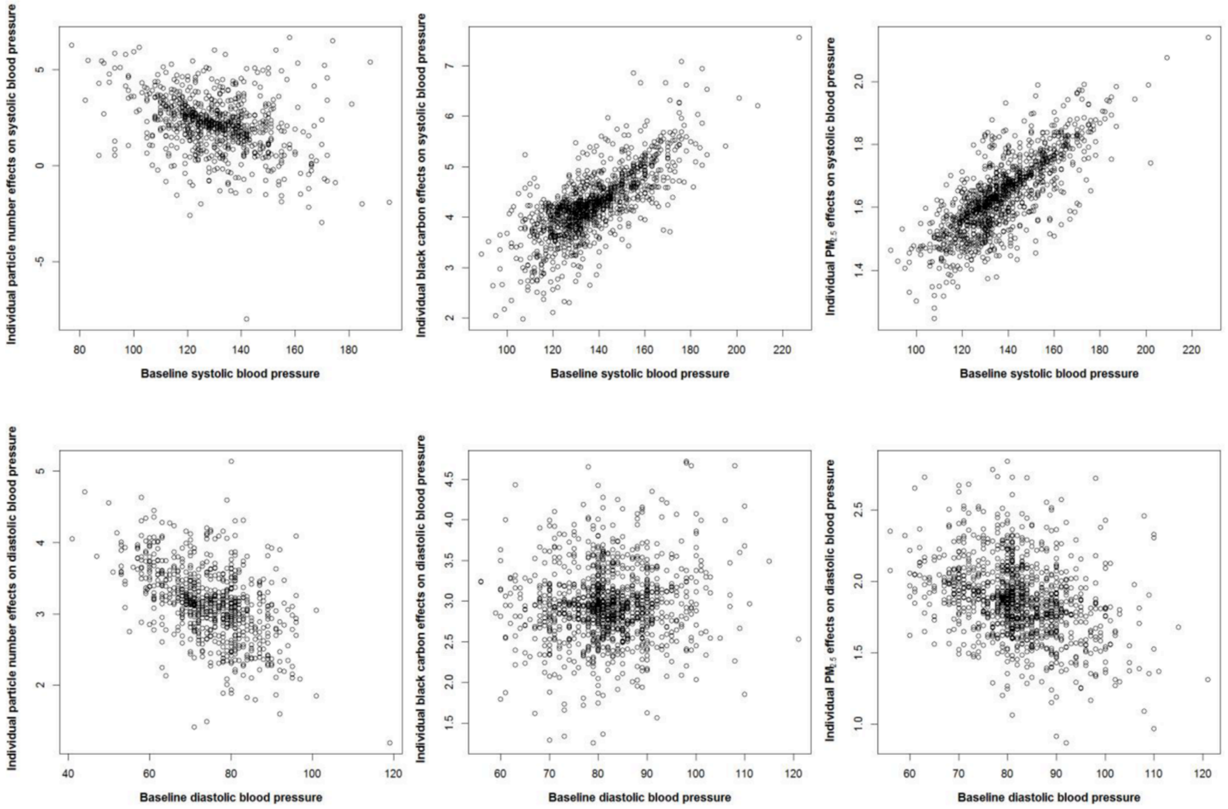

Figure S1. Individual effects of traffic-related air pollutants on systolic blood pressure and diastolic blood pressure versus baseline systolic blood pressure and baseline diastolic blood pressure, respectively. We fit linear mixed-effects models with random intercepts and slopes for individual air pollutant effects to check for heterogeneous associations with the same outcomes of interest. We adjusted for temperature, relative humidity, sine and cosine terms of the days of the season, age, physician-diagnosed diabetes, body mass index, smoking status, cumulative cigarette pack-years, current use of statin, and current use of antihypertensive medications. Conditional on algorithm convergence, we obtained the subject-specific random slopes and calculated the individual effects (by adding the fixed and random effects) for participants with more than one visit. Subsequently, we plotted these individual effects (that correspond to individual increases in the outcome for an interquartile range increase in exposure) versus the outcome of interest measured at baseline. The units of the y-axes correspond the units of the outcome. Estimates are limited to participants with more than one study visit.

Figure S2

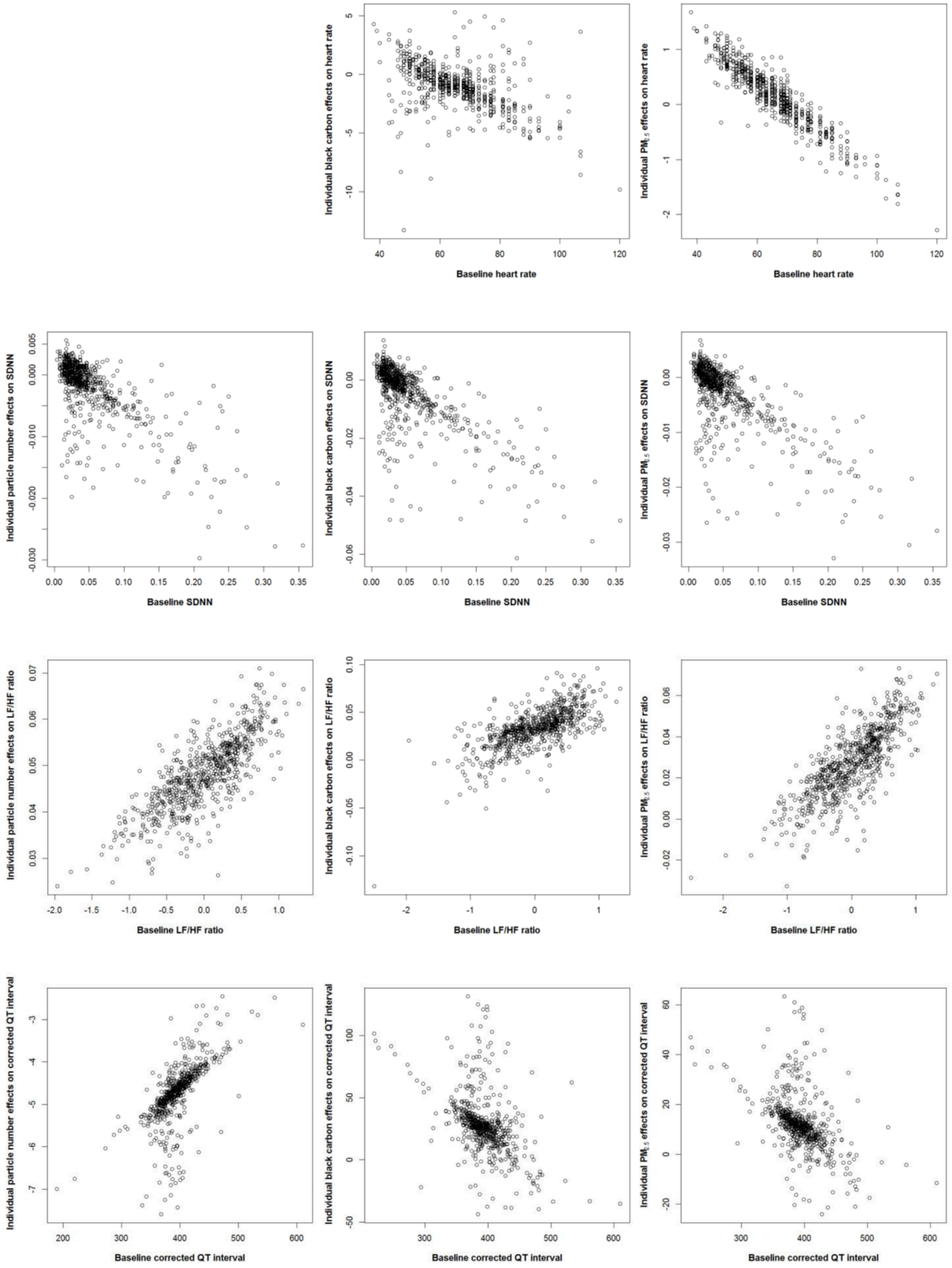

Individual black carbon effects on heart rate
Baseline heart rate
Individual PM2.5 effects on heart rate
Baseline heart rate
Individual particle number effects on SDNN
Baseline SDNN
Individual black carbon effects on SDNN
Baseline SDNN
Individual PM2.5 effects on SDNN
Baseline SDNN
Individual particle number effects on LF/HF ratio
Baseline LF/HF ratio
Individual black carbon effects on LF/HF ratio
Baseline LF/HF ratio
Individual PM2.5 effects on LF/HF ratio
Baseline LF/HF ratio
Individual particle number effects on corrected QT interval
Baseline corrected QT interval
Individual black carbon effects on corrected QT interval
Baseline corrected QT interval
Individual PM2.5 effects on corrected QT interval
Baseline corrected QT interval

Figure S2. Individual effects of traffic-related air pollutants on heart rate, SDNN, log(LF/HF), and corrected QT interval versus baseline heart rate, baseline SDNN, baseline log(LF/HF), and baseline corrected QT interval, respectively. We fit linear mixed-effects models with random intercepts and slopes for individual air pollutant effects to check for heterogeneous associations with the same outcomes of interest. We adjusted for temperature, relative humidity, sine and cosine terms of the days of the season, age, physician-diagnosed diabetes, body mass index, smoking status, cumulative cigarette pack-years, current use of statin, and current use of antihypertensive medications. For SDNN, we also controlled for heart rate because standard deviation is likely to be larger as heart rate increases. Conditional on algorithm convergence, we obtained the subject-specific random slopes and calculated the individual effects (by adding the fixed and random effects) for participants with more than one visit. Subsequently, we plotted these individual effects (that correspond to individual increases in the outcome for an interquartile range increase in exposure) versus the outcome of interest measured at baseline. The units of the y-axes correspond the units of the outcome. Estimates are limited to participants with more than one study visit.

Figure S3

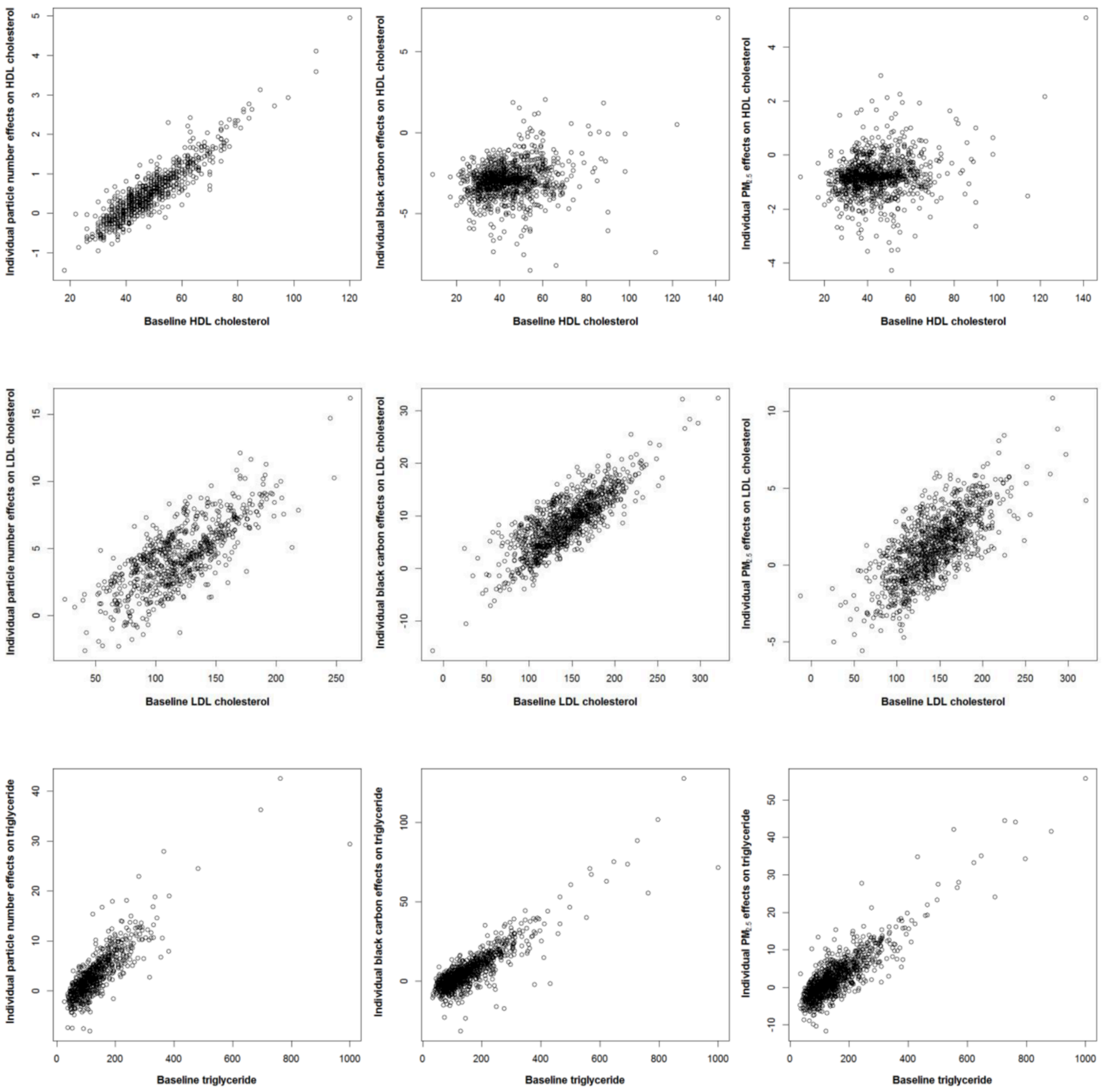

Figure S3. Individual effects of traffic-related air pollutants on HDL cholesterol, LDL cholesterol, and triglyceride versus baseline HDL cholesterol, baseline LDL cholesterol and baseline triglyceride, respectively. We fit linear mixed-effects models with random intercepts and slopes for individual air pollutant effects to check for heterogeneous associations with the same outcomes of interest. We adjusted for temperature, relative humidity, sine and cosine terms of the days of the season, age, physician-diagnosed diabetes, body mass index, smoking status,

cumulative cigarette pack-years, and current use of statin. Conditional on algorithm convergence, we obtained the subject-specific random slopes and calculated the individual effects (by adding the fixed and random effects) for participants with more than one visit. Subsequently, we plotted these individual effects (that correspond to individual increases in the outcome for an interquartile range increase in exposure) versus the outcome of interest measured at baseline. The units of the y-axes correspond the units of the outcome. Estimates are limited to participants with more than one study visit.

Figure S4

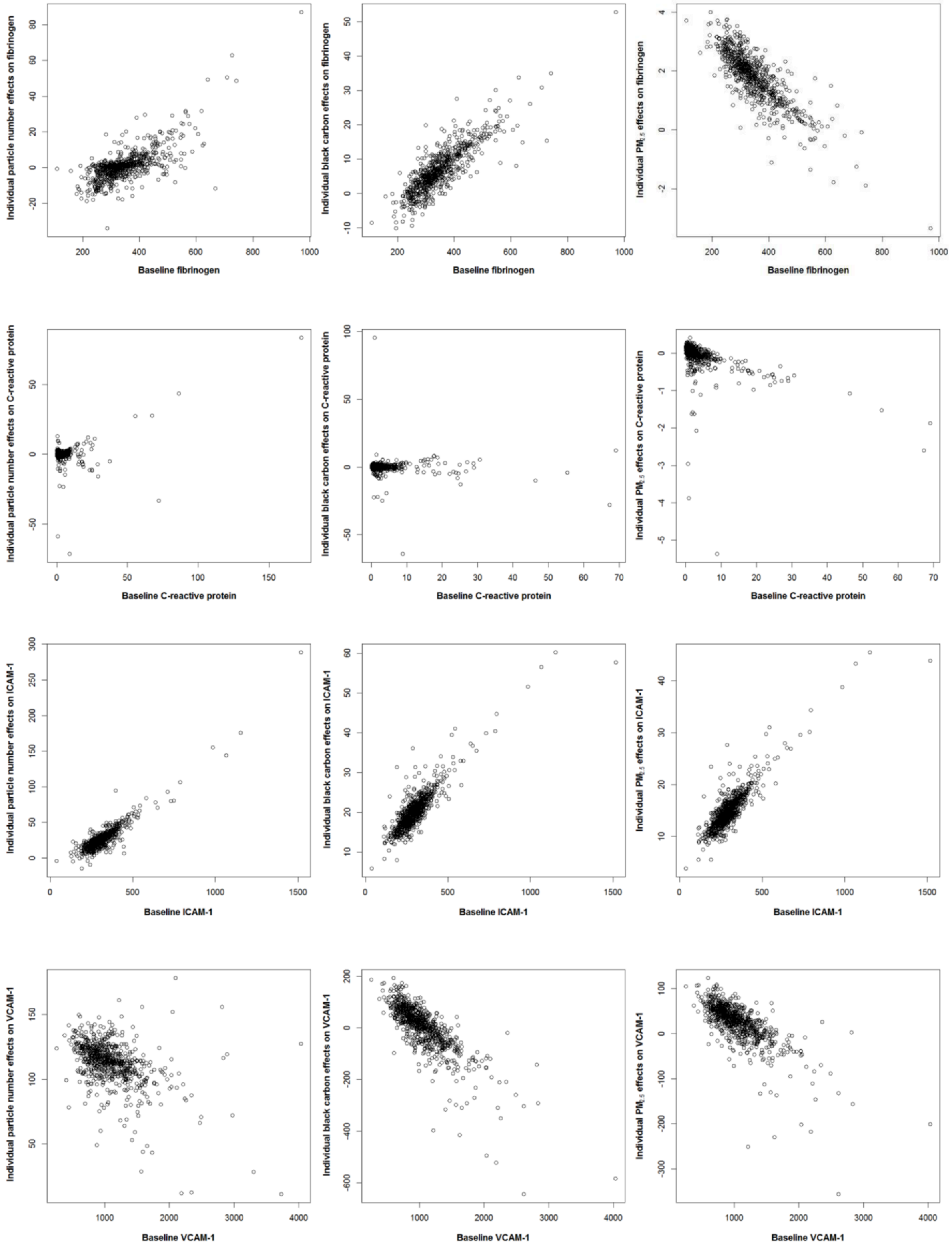
Individual particle number effects on fibrinogen
Individual black carbon effects on fibrinogen
Individual $PM_{2.5}$ effects on fibrinogen
Baseline fibrinogen
Baseline fibrinogen
Baseline fibrinogen
Individual particle number effects on C-reactive protein
Individual black carbon effects on C-reactive protein
Individual $PM_{2.5}$ effects on C-reactive protein
Baseline C-reactive protein
Baseline C-reactive protein
Baseline C-reactive protein
Individual particle number effects on ICAM-1
Individual black carbon effects on ICAM-1
Individual $PM_{2.5}$ effects on ICAM-1
Baseline ICAM-1
Baseline ICAM-1
Baseline ICAM-1
Individual particle number effects on VCAM-1
Individual black carbon effects on VCAM-1
Individual $PM_{2.5}$ effects on VCAM-1
Baseline VCAM-1
Baseline VCAM-1
Baseline VCAM-1

Figure S4. Individual effects of traffic-related air pollutants on fibrinogen, C-reactive protein, ICAM-1, and VCAM-1 versus baseline fibrinogen, baseline C-reactive protein, baseline ICAM-1, and baseline VCAM-1, respectively. We fit linear mixed-effects models with random intercepts and slopes for individual air pollutant effects to check for heterogeneous associations with the same outcomes of interest. We adjusted for temperature, relative humidity, sine and cosine terms of the days of the season, age, physician-diagnosed diabetes, body mass index, smoking status, cumulative cigarette pack-years, and current use of statin. Conditional on algorithm convergence, we obtained the subject-specific random slopes and calculated the individual effects (by adding the fixed and random effects) for participants with more than one visit. Subsequently, we plotted these individual effects (that correspond to individual increases in the outcome for an interquartile range increase in exposure) versus the outcome of interest measured at baseline. The units of the y-axes correspond the units of the outcome. Estimates are limited to participants with more than one study visit.
